# Clinical characteristics of myasthenia gravis patients with coexistence of AChR and titin antibodies

**DOI:** 10.3389/fneur.2025.1679404

**Published:** 2025-11-28

**Authors:** Siqi Wang, Difang Shi, Shanshan Wu, Qijie Zhang, Weiwei Xiao, Fang Qian, Yan Zheng, Jianjian Bao, Jia Liu, Xujun Chu, Kang Du

**Affiliations:** 1 Department of Clinical Pharmacy, Yunnan Qujing Central Hospital (Qujing First People’s Hospital), Qujing, Yunnan, China; 2 Department of Thoracic Surgery I, The Third Affiliated Hospital of Kunming Medical University, Yunnan Cancer Hospital, Peking University Cancer Hospital Yunnan, Kunming, Yunnan, China; 3 Department of Neurology, Qujing Second People’s Hospital, Qujing, Yunnan, China; 4 Department of Neurology, Yunnan Qujing Central Hospital (Qujing First People’s Hospital), Qujing, Yunnan, China; 5 Department of Neurology, The Affiliated Shandong Provincial Hospital of Shandong First Medical University, Jinan, Shandong, China

**Keywords:** myasthenia gravis, AChR and titin antibody, thyroid function, thymoma, Southwest China

## Abstract

**Objective:**

This study aimed to investigate the clinical characteristics of patients with myasthenia gravis (MG) who are double-seropositive for acetylcholine receptor antibodies (AChR-Ab^+^) and titin antibodies (Titin-Ab^+^).

**Methods:**

A retrospective analysis was conducted on MG patients hospitalized in the Department of Neurology between March 2020 and June 2024. Patients were categorized into two groups based on antibody profiles: those with AChR antibody positivity alone and those with dual positivity for AChR and titin antibodies. Clinical features, MG classification, and treatment outcomes were compared between the two groups.

**Results:**

A total of 35 MG patients were included, comprising 18 with single AChR-Ab^+^ and 17 with AChR-Ab^+^/Titin-Ab^+^. The dual antibody-positive group showed a significantly higher proportion of patients with MGFA classification above Class II (47.1%) and a higher rate of thyroid dysfunction (50%) compared to the single antibody-positive group (*p* = 0.001). The median age of onset in the dual antibody-positive group was older than that in the single antibody-positive group (67.0 years vs. 58.5 years), although this difference was not statistically significant (*p* > 0.05). No significant differences were observed between the two groups in terms of gender, initial symptoms, clinical manifestations, thymic hyperplasia, proportion of thymoma, treatment regimens, or therapeutic outcomes (all *p* > 0.05).

**Conclusion:**

This study provides the first systematic characterization of the clinical profile of AChR-Ab^+^/Titin-Ab^+^ myasthenia gravis patients in Southwest China. Our findings indicate that dual antibody-positive MG patients are more prone to generalized disease involvement and have a higher susceptibility to thyroid dysfunction.

## 1 Introduction

MG is a chronic autoimmune disorder characterized by impaired neuromuscular transmission due to autoantibody-mediated damage at the neuromuscular junction, primarily manifesting as skeletal muscle fatigability and weakness (1). Commonly affected muscle groups include ocular, facial, masticatory, pharyngeal, cervical, and proximal limb muscles, with potential respiratory involvement causing dyspnea in severe cases (2). To date, several pathogenic MG-associated autoantibodies have been identified, including acetylcholine receptor antibodies (AChR-Ab), muscle-specific tyrosine kinase receptor antibodies (MuSK-Ab), low-density lipoprotein receptor-related protein 4 antibodies (LRP4-Ab), titin antibodies (Titin-Ab), and antibodies against the ryanodine receptor calcium release channel (RyR-Ab) (3, 4). Among these, AChR-Ab represents the most prevalent pathogenic antibody, detectable in approximately 80–90% of patients with generalized MG (5, 6). Recent studies have shown that AChR-Ab can mediate neuromuscular junction impairment, thereby causing muscle weakness (7, 8). Moreover, the Titin-Ab, is frequently observed in MG patients with thymoma, particularly in those with late-onset disease, demonstrating a higher positivity rate (9–12). Subsequently, accumulating evidence indicates that the presence of Titin-Ab suggests concomitant thymoma, more severe clinical manifestations, an increased requirement for immunosuppressive therapy, compared to patients positive for AChR-Ab alone (12–14).

Notably, some studies have demonstrated that patients with AChR-Ab^+^/Titin-Ab^+^ usually exhibit a later age of onset and a more rapid progression to generalized MG. These patients frequently present with more severe bulbar dysfunction, higher clinical severity scores, and an increased incidence of thymoma (15). However, most current research focused on the immunopathological mechanisms and treatment strategies associated with single antibody subtypes, with limited comprehensive clinical data available for the AChR-Ab^+^/Titin-Ab^+^ subgroup. This study aimed to characterize the clinical profile of AChR-Ab^+^/Titin-Ab^+^ myasthenia gravis (MG) in Southwest China, with a focus on patterns of muscle involvement, disease severity, thyroid function, and thymic pathology including thymoma, which is of critical importance for optimizing stratified management and individualized therapeutic approaches in MG.

## 2 Materials and methods

A retrospective analysis was conducted on the clinical data of patients newly diagnosed with MG for the first time between March 2020 and June 2024 at the Department of Neurology, Qujing First People’s Hospital. The inclusion and exclusion criteria were as follows. Inclusion criteria: (1) Diagnosis met the criteria outlined in the *Chinese Guidelines for the Diagnosis and Treatment of Myasthenia Gravis (2020 Edition)* (16), which include fluctuating muscle weakness with fatigability and at least two positive results among the following: serum antibody testing, neostigmine test, and repetitive nerve stimulation (RNS); (2) Patients with newly diagnosed MG; (3) Adult-onset cases presenting with fluctuating muscle weakness, typically worse in the evening; (4) Completion of diagnostic evaluations, including neostigmine test, chest computed tomography (CT) for thymic assessment, RNS, and serum antibody testing for MG-related antibodies; and (5) Positive serum antibody results indicating either: (i) MG patients with acetylcholine receptor antibody positivity (AChR-Ab^+^); or (ii) MG patients with both acetylcholine receptor antibody and titin antibody positivity (AChR-Ab^+^/Titin-Ab^+^). Exclusion criteria: (1) Severe cardiac or pulmonary insufficiency; (2) Significant sequelae of major illnesses that may affect clinical evaluation; (3) MG patients positive for MuSK-Ab, RyR-Ab, or other related antibodies; and (4) Incomplete clinical data. In this study, patients were screened according to strict inclusion and exclusion criteria. Initially, 65 patients meeting the diagnostic criteria for myasthenia gravis were screened. Among these, 30 patients were excluded due to incomplete data, lack of serum-specific antibody testing, or presence of other specific antibodies. Finally, 35 MG patients positive for AChR-Ab^+^ with or without Titin-Ab^+^ were included in this study. This was a single-center retrospective study, collected data included: sex, age at disease onset (with 50 years as the cutoff between early-onset and late-onset), initial symptoms, clinical manifestations (including affected muscle groups and Myasthenia Gravis Foundation of America (MGFA) clinical classification: Class I–V), disease duration, presence of myasthenic crisis, thymic status, thyroid function, ocular MG (OMG) progression to generalized MG (GMG), RNS findings, neostigmine test results, treatment regimen (including cholinesterase inhibitors, corticosteroids, and immunosuppressants), and therapeutic outcomes.

Laboratory assessments included the detection of serum MG-specific antibodies: AChR-Ab, MuSK-Ab, Titin-Ab, RyR-Ab, and LRP4-Ab. These antibodies were measured using a cell-based assay (CBA). In the CBA method, conventional fluorescent secondary antibodies were used as detection signals. Antibody titers were interpreted as follows: weak positive (+) at 1:10, moderately positive (++) at 1:100, and strongly positive (+++) at 1:1000 (17). For thyroid function evaluation, venous blood was collected from all patients to measure thyroid stimulating hormone (TSH), total triiodothyronine (TT3), total thyroxine (TT4), free triiodothyronine (FT3), free thyroxine (FT4), thyroid dysfunction was defined as abnormal elevation or reduction in TSH, FT4, or FT3 levels. Thyroid dysfunction status was determined by a comprehensive assessment based on the documented past medical history in the admission records and the initial thyroid function test results obtained during the current hospitalization.

## 3 Statistical analysis

Continuous variables were presented as median (percentile). The continuous variables showed an abnormal distribution (as evaluated by single sample K-S test), and Mann–Whitney U test was used for evaluating differences of continuous variables between two groups. The categorical data were expressed as the number of cases or the constituent ratio (%), and Chi-square test or Fisher’s exact test was applied. Two-sided *p* values were calculated for all analyses; *p* < 0.05 was considered significant. Statistical analysis was performed with SPSS Statistics, version 24 (IBM, Armonk, NY, United States).

## 4 Results

### 4.1 Descriptive analysis of demographic characteristics

Clinical data from a total of 35 patients with MG were collected, including 18 males and 17 females. Based on serum antibody profiles, patients were categorized into two groups: those positive for AChR-Ab^+^ (*n* = 18, male: female = 9: 9) and those positive for both AChR-Ab^+^/Titin-Ab^+^ (*n* = 17, male: female = 9: 8). No statistically significant difference in sex distribution was observed between the two groups. The median age of disease onset in the AChR-Ab^+^/Titin-Ab^+^ group was older (67.0 years) compared to the AChR-Ab^+^ group (58.5 years); however, this difference was not statistically significant (*p* = 0.08).

Due to the limited sample size in this study, with comparable numbers in the AChR-Ab^+^/Titin-Ab^+^ group and AChR-Ab^+^ group, a comparison between early- and late-onset subgroups was not performed. The small subgroup sizes would have resulted in limited statistical power, rendering *p*-values potentially uninterpretable. Therefore, odds ratios and Cohen’s d values were supplemented to assess clinical relevance. Detailed results are presented in Table 1.

**Table 1:** Comparison of clinical characteristics between AChR-Ab^+^ and AChR-Ab^+^/Titin-Ab^+^ MG patients.

Parameter	AChR-Ab^+^ (*n* = 18)	AChR-Ab^+^/Titin-Ab^+^ (*n* = 17)	*p*	OR (95%CI)	Cohen’s d (95%CI)
Sex, male, *n* (%)	9 (50)	9 (52.9)	>0.99	1.13 (0.298–4.241)	NA
Age, M (IQR)	58.50 (37.75, 62.50)	67.00 (50.50, 72.00)	0.08	NA	0.60 (−0.09, 1.29)
Initial symptoms, *n* (%)					
Early onset	6 (33.3)	4 (23.5)	0.71	0.62 (0.139–2.727)	NA
Extraocular muscles	16 (88.9)	16 (94.1)	>0.99	2.00 (0.170–23.542)	NA
Bulbar muscles	3 (16.7)	7 (41.2)	0.15	3.50 (0.783–15.632)	NA
Neck muscles	2 (11.1)	0 (0)	0.49	0.19 (0.009–4.068)^a^	NA
Respiratory muscles	0 (0)	0 (0)	>0.99	NA	NA
Limbs	4 (22.2)	2 (11.8)	0.66	0.47 (0.074–2.959)	NA
Clinical manifestations (involved muscle groups), *n* (%)					NA
External ophthalmoplegia	16 (88.9)	16 (94.10)	>0.99	2.00 (0.165–24.322)	NA
Bulbar muscles	4 (22.2)	8 (47.1)	0.16	3.11 (0.721–13.420)	NA
Neck muscles	2 (11.1)	2 (11.8)	>0.99	1.07 (0.126–9.018)	NA
Respiratory muscles	2 (11.1)	6 (35.3)	0.12	4.37 (0.815–23.336)	NA
Limbs	2 (11.1)	4 (23.5)	0.36	2.47 (0.394–15.397)	NA
Ocular MG	10 (55.6)	6 (35.3)	0.31	0.44 (0.109–1.744)	NA
Generalized MG	8 (44.4)	10 (58.8)	0.51	1.79 (0.521–6.117)	NA
MGFA classification, *n* (%)					NA
Class I	11 (61.1)	6 (35.3)	0.18	0.35 (0.094–1.285)	NA
Class II	7 (38.9)	3 (17.7)	0.26	0.34 (0.072–1.570)	NA
Class III	0 (0.0)	6 (35.3)	0.008**	19.64 (0.996–386.8)^a^	NA
Class IV	0 (0.0)	0 (0.0)	>0.99	NA	NA
Class V	0 (0.0)	2 (11.8)	0.23	4.80 (0.232–99.225)^a^	NA
Class >II	0 (0.0)	8 (47.1)	0.001**	33.00 (1.742–625.4)^a^	NA
OMG to GMG conversion	1 (5.6)	2 (11.8)	0.60	2.27 (0.184–27.838)	NA
Thymic status, *n* (%)					NA
Thymoma	6 (42.9)^b^	3 (21.4)^b^	0.42	0.37 (0.075–1.756)	NA
Normal thymus	5 (35.7)^b^	9 (64.3)^b^	0.26	3.24 (0.739–14.218)	NA
Thymectomy	2 (14.3)^b^	3 (21.4)^b^	>0.99	1.64 (0.236–11.343)	NA
Thymic hyperplasia	3 (21.4)^b^	2 (14.3)^b^	>0.99	0.61 (0.088–4.244)	NA
Thyroid dysfunction	1 (6.7)^c^	7 (50.0)^b^	0.01*	14.00 (1.531–128.071)	NA
Positive RNS	10 (83.3)^d^	10 (83.3%)^d^	>0.99	1.00 (0.128–7.823)	NA
Treatment, *n* (%)					NA
Pyridostigmine	18 (100.0)	14 (100.0)	>0.99	NA	NA
Glucocorticoids	11 (61.1)	13 (76.5)	0.47	2.07 (0.524–8.159)	NA
Immunosuppressants	5 (27.8)	6 (35.3)	0.72	1.42 (0.353–5.693)	NA
Treatment response, *n* (%)					NA
Significant improvement	15 (83.3)	14 (82.4)	>0.9999	0.93 (0.202–4.304)	NA
Improvement	3 (16.7)	2 (11.8)	>0.9999	0.67 (0.094–4.722)	NA
Worsening	0 (0.0)	1 (5.9)	0.49	2.28 (0.089–58.143) ^a^	NA

M (IQR), Interquartile Range (25th percentile, 75th percentile); NA, not available; OR, odds ratios.

a OR estimated with Haldane–Anscombe continuity correction (0.5 added to each cell) because of zero event(s) in one arm.

b The number of MG patients included was based on the subsample size, *n* = 14.

c The number of patients included was based on the subsample size, *n* = 15.

d The number of patients included was based on the subsample size, *n* = 12.

Values in bold indicate a statistically significant difference (*p* < 0.05).

### 4.2 Initial symptoms and clinical manifestations

In terms of initial symptoms, within the AChR-Ab^+^ group, there were 6 cases of early-onset MG, with initial involvement of the extraocular muscles in 16 cases, bulbar muscles in 3 cases, cervical muscles in 2 cases, and limb muscles in 4 cases. In the AChR-Ab^+^/Titin-Ab^+^ group, 4 patients had early-onset MG, with initial symptoms involving the extraocular muscles in 16 cases, bulbar muscles in 7 cases, and limb muscles in 2 cases; no cases had cervical muscle onset. Neither group presented with respiratory muscle involvement at disease onset. The proportion of MG patients with bulbar onset was higher in the AChR-Ab^+^/Titin-Ab^+^ group (41.2%) than those in the AChR-Ab^+^ group (16.7%), although the difference was not statistically significant (*p* = 0.15). Overall, there were no statistically significant differences in initial symptom distribution between the two groups.

Regarding clinical manifestations (muscle groups involved), no significant differences were observed between the two groups in the involvement of extraocular, bulbar, cervical, respiratory, or limb muscles. In the AChR-Ab^+^ group, 10 patients were classified as having ocular MG, while 8 had generalized MG; in the AChR-Ab^+^/Titin-Ab^+^ group, 6 patients had ocular MG and 10 had generalized MG. There were no statistically significant differences in the distribution of affected muscle groups between the two groups (*p* > 0.05).

### 4.3 MGFA classification

**Figure 1:**
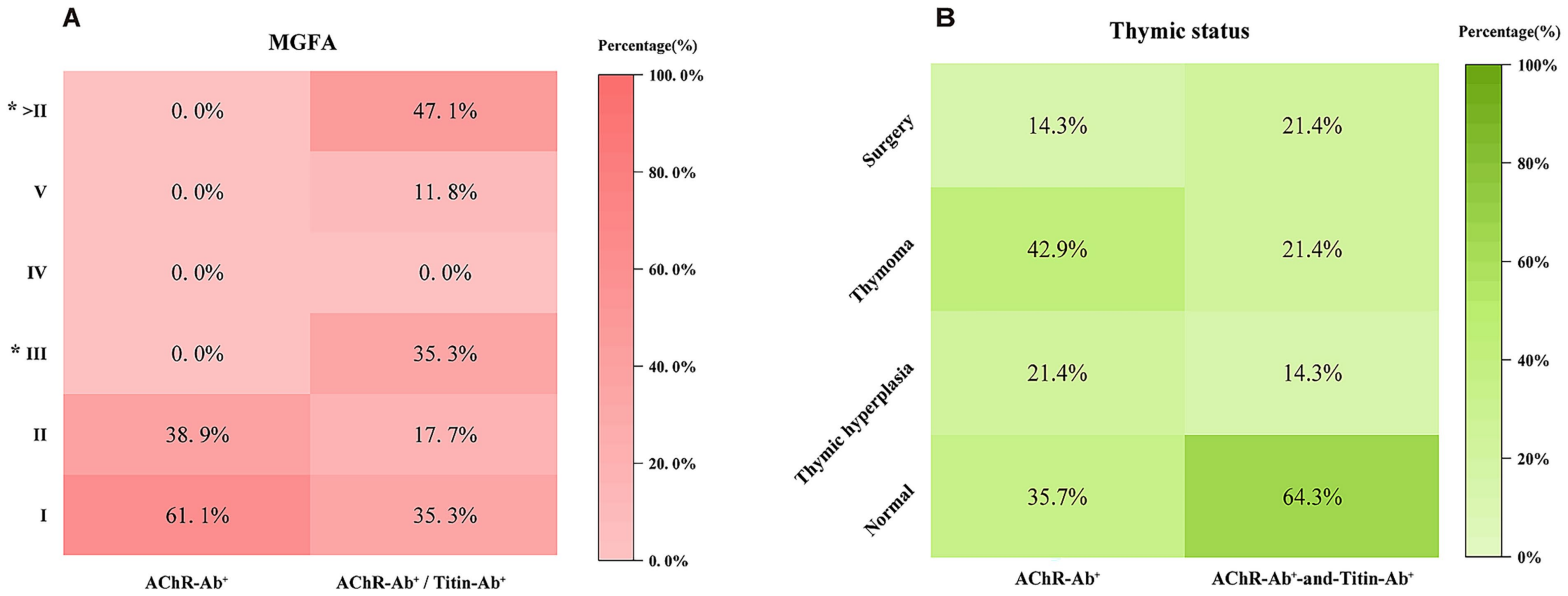
**(A)** Distribution of MGFA clinical classifications in patients with AChR-Ab^+^ versus AChR-Ab^+^/Titin-Ab^+^. The heatmap illustrates the proportion of each MGFA class. Y-axis labels represent MGFA clinical classes, “>II” indicates classifications more severe than Class II. “*” Indicates a significant difference between single- and double-antibody groups (*p* < 0.05); **(B)** Distribution of thymic histopathology and concomitant thyroid dysfunction in AChR-Ab^+^ versus AChR-Ab^+^/Titin-Ab^+^ patients. The heatmap displays the proportion of each thymic category, arranged by pathological progression (Normal → Hyperplasia → Thymoma → Thymectomy), and the prevalence of thyroid dysfunction. “*” Indicates a significant difference between the two groups (*p* < 0.05).

According to the MGFA classification, in the AChR-Ab^+^ group, 11 patients were classified as Class I and 7 as Class II; no patients were classified as > Class II (i.e., Class III, IV, or V). In contrast, within the AChR-Ab^+^/Titin-Ab^+^ group, 6 patients were classified as Class I, 3 as Class II, 6 as Class III, and 2 as Class V, resulting in a total of 8 patients classified as > Class II. The proportion of patients in Class III was significantly higher in the AChR-Ab^+^/Titin-Ab^+^ group than in the AChR-Ab^+^ group (*p* = 0.008). The difference in the proportion of patients classified as > Class II between the two groups was also statistically significant (*p* = 0.001). The distribution of MGFA clinical classifications shows that double-antibody patients exhibit a higher proportion of severe MGFA classes (Figure 1A).

### 4.4 Thymus status and thyroid function

No significant differences were observed between the two groups in terms of thymoma incidence, normal thymus, thymectomy, thymic hyperplasia, or positive results from repetitive nerve stimulation (RNS) testing. However, the proportion of patients with abnormal thyroid function was significantly higher in the AChR-Ab^+^/Titin-Ab^+^ group (7/17) compared to the AChR-Ab^+^ group (*p* = 0.01), indicating statistical significance. Notably, in the AChR-Ab^+^/Titin-Ab^+^ group, the ratio of hyperthyroidism to hypothyroidism was 2:9; whereas in the AChR-Ab^+^ group, the ratio was 1:14. Thymic status across the two groups, highlighting the prevalence of thymoma, thymic hyperplasia, and normal thymus, is also depicted (Figure 1B).

### 4.5 Treatment and therapeutic efficacy

There were no significant differences between the two groups in the use of pyridostigmine bromide, corticosteroids, immunosuppressive agents, or in overall treatment efficacy (*p* > 0.05). Detailed treatment outcomes are provided in Table 1.

## 5 Discussion

Titin, functions primarily as a scaffolding molecule essential for maintaining sarcomeric integrity. In the context of aberrant immune responses against muscle tissue, Titin-Ab may erroneously target and damage muscle cells, leading to structural disruption (18); Titin-Ab specifically recognizes the MGT-30 antigen, which is encoded by the cDNA sequence at the A/I junctional region of the titin protein, this interaction interferes with normal neuromuscular transmission and sarcomere assembly, thereby exacerbating the severity of MG (19). Our study found that higher MGFA class and thyroid dysfunction in AChR-Ab^+^/Titin-Ab^+^ MG patients compared to the AChR-Ab^+^ MG patients.

The presence of Titin-Ab in patients with MG has been associated with factors such as age of onset, presence of thymoma, and disease severity. For instance, Titin-Ab are more frequently detected in patients aged ≥ 60 years, but are relatively uncommon in younger individuals, suggesting a potential association between Titin-Ab positivity and late-onset myasthenia gravis (LOMG) (20, 21). In our study, the median age of onset in AChR-Ab^+^/Titin-Ab^+^ MG patients were higher than that in those with AChR-Ab^+^ alone, with the difference approaching statistical significance, further supporting the hypothesis that Titin-Ab may serve as a biomarker for LOMG. These findings are consistent with those of Suzuki (19) and Dai Junjie (22). Clinical studies have shown that titin antibody testing has a sensitivity of 69% and a specificity of 97% for thymic epithelial tumors, and Titin-Ab are detectable in approximately 70 to 90% of MG patients with thymoma (23, 24). However, a causal relationship between Titin-Ab and thymoma has not yet been clearly established. In our study, there was no significant difference in the prevalence of thymoma between the AChR-Ab^+^/Titin-Ab^+^ group and AChR-Ab^+^ group; paradoxically, the AChR-Ab^+^ group had a higher proportion of thymoma. Possible explanations include small sample size (*n* = 14), population differences, or age-related factors. However, most AChR-Ab^+^-and-Titin-Ab^+^ patients in our study were aged >60 years, consistent with the lower thymoma prevalence typically observed in LOMG.

Titin-Ab plays an important clinical role in the pathogenesis and management of MG. For instance, Romi F et al. demonstrated a correlation between disease severity and Titin-Ab titer in individual MG patients. Titin-Ab was detected significantly more frequently in patients with severe MG than in those with milder disease, indicating that the presence of this antibody is associated with more severe disease within MG subgroups (2, 25). Sieb et al. (26) similarly demonstrated that Titin-Ab^+^ patients often exhibit more severe clinical symptoms and poorer prognosis, with positivity rates rising from 38 to 100% as disease severity increases. Our findings further support this trend: patients with single antibody positivity were predominantly classified as MGFA class I–II, with no cases of moderate-to-severe generalized MG (MGFA III–V), whereas 47.1% of dual-positive patients were classified as MGFA III–V, including 35.3% as MGFA III and 11.8% as MGFA V. These findings suggest that the seropositivity rate for Titin-Ab becomes increasingly prevalent with increasing clinical severity of MG. Similar findings were reported by Suzuki et al. further supporting the link between Titin-Ab and more severe MG phenotypes (27). Additionally, evidence suggests that MG patients may develop different autoantibodies at various disease stages: AChR-Ab tends to dominate in early stages, while skeletal muscle–related antibodies, including Titin-Ab and anti-ryanodine receptor antibodies, may emerge with disease progression (28). This may be attributed to structural damage to muscle cells induced by Titin-Ab, which impairs sarcomere assembly and consequently leads to the involvement of extensive muscle groups (29). Taken together, Titin-Ab may be associated with MG disease severity, being most frequently detected in severe cases and serving as a biomarker for disease severity in MG patients. Therefore, testing for Titin-Ab should be considered during the initial management of MG.

To evaluate the severity of clinical manifestations in patients positive for AChR-Ab^+^/Titin-Ab^+^, we analyzed the initial symptoms and disease progression in comparison to those with AChR-Ab^+^ alone. Both groups primarily presented with extraocular muscle involvement at onset; however, the AChR-Ab^+^/Titin-Ab^+^ group demonstrated a higher proportion of bulbar muscle involvement as the initial symptom. Clinically, respiratory muscle involvement was significantly more prevalent in the AChR-Ab^+^/Titin-Ab^+^ group, suggesting an increased risk of respiratory failure in this subgroup. Therefore, heightened vigilance for respiratory compromise is warranted in MG patients with concurrent AChR-Ab^+^/Titin-Ab^+^. Current common treatments for MG include acetylcholinesterase (AChE) inhibitors, immunosuppressive medications, thymectomy, intravenous immunoglobulin (IVIG), and plasmapheresis. Yu-Hong et al. through analysis of 437 Chinese adult MG patients, found that MG patients with positive Titin-Ab often present with more severe disease and require more aggressive immunosuppressive therapy (30). Cordts et al. previously reported that MG patients positive for both AChR-Ab^+^/Titin-Ab^+^ require long-term use of immunosuppressants therapy compared to those with only AChR-Ab^+^ (31). In contrast, our findings showed no statistically significant difference in the use of immunosuppressive agents between the two groups. Moreover, both groups exhibited clinical improvement following immunotherapy. The vast majority of MG patients, regardless of their antibody status, show varying degrees of response to immunotherapy (32). Among these, the utilization rate of immunosuppressants was the highest, and combination therapy with corticosteroids and immunosuppressants demonstrated superior efficacy (33). Recent studies have demonstrated favorable efficacy of the monoclonal antibody rituximab (RTX) in patients with myasthenia gravis refractory to standard therapy (34). A related case report also described an AChR-Ab^+^/Titin-Ab^+^ MG patient who showed significant clinical improvement after treatment with Teltacicept, following an inadequate response to conventional therapies (18).

MG is frequently associated with other autoimmune disorders, with thyroid disease being significantly more prevalent in MG patients compared to the general population (35). Mu et al. reported that the incidence of thyroid dysfunction in MG patients with AChR-Ab^+^ was as high as 100%, and the prevalence of thyroid abnormalities also approached 100% in those with the AChR-Ab^+^/Titin-Ab^+^ MG patients (36). Similarly, Toth et al. demonstrated that thyroid dysfunction is commonly observed in AChR-Ab^+^ MG patients. Both MG and autoimmune thyroid diseases are organ-specific, antibody-mediated disorders and have significant clinical overlap (37). Gilhus et al. also confirmed a significantly increased incidence of both organ-specific and systemic autoimmune diseases, especially thyroiditis, in patients with ocular MG (38). Multiple large-scale, multicenter studies have confirmed that the overall incidence of thyroid dysfunction in MG patients ranges from 10 to 30%, significantly higher than that in the general population. Moreover, the greater the diversity of autoantibodies present in MG patients (e.g., concurrent presence of AChR, Titin, and MuSK antibodies), the higher the incidence of thyroid dysfunction, among which Hashimoto’s thyroiditis is the most common and often progresses to hypothyroidism. MG and thyroid dysfunction (including hyperthyroidism, hypothyroidism, and subclinical thyroid abnormalities) are closely associated, sharing a common autoimmune pathogenesis. Structural similarities between thyroid tissue antigens (such as thyroid peroxidase and thyroglobulin) and neuromuscular junction antigens (e.g., acetylcholine receptor, AChR) may trigger “cross-reactive immune responses,” leading to simultaneous immune attacks on both the thyroid gland and the skeletal muscle postsynaptic membrane (39). Our study also found that the incidence of thyroid dysfunction was significantly higher in the AChR-Ab^+^/Titin-Ab^+^ group compared to the AChR-Ab^+^ group, while the thyroid dysfunction in both groups was predominantly characterized by hypothyroidism. A plausible pathophysiological mechanism is that the presence of both AChR-Ab and Titin-Ab may exacerbate autoimmune dysregulation in MG, potentially promoting an immune response that extends to thyroid tissue and leads to its immunological attack (40). Additionally, autoimmune thyroid disorders are characterized not only by thyroid-specific antigen targeting but also by a broader systemic immune activation, which may contribute to the generation of neuromuscular junction antibodies, including AChR-Ab and Titin-Ab (41). Ariadna Z et al. (42) found that thyroid hormones significantly influence skeletal muscle function, with possible manifestations such as myalgia and muscle weakness, their study also suggested that titin might serve as a novel marker for musculoskeletal symptom exacerbation in thyroid disease. This may partly explain the more severe clinical symptoms observed in the AChR-Ab^+^/Titin-Ab^+^ group compared to the AChR-Ab^+^ group. Given the close association between thyroid disorders and MG, routine screening for thyroid dysfunction is warranted in all patients diagnosed with MG.

Our study has several limitations. This was a retrospective, single-center study with a small sample size (*n* = 35), which may limit the generalizability of the findings. Additionally, the study lacked longitudinal follow-up after patient discharge and quantitative antibody titer data. Regarding treatment, this study only compared differences among commonly used conventional therapies, without providing detailed information on individual patient treatment regimens. Therefore, furthermore, existing literature has not yet thoroughly documented differences in treatment strategies between double antibody-positive and single antibody-positive MG patients. Future studies should expand the sample size and employ multicenter, prospective designs to further validate the associations between thymoma, thyroid function, and dual antibody positivity. In addition, quantitative scoring of MGFA clinical classifications should be performed, novel targeted therapeutic regimens should be explored and compared, and detailed records of patient treatment strategies should be maintained, accompanied by long-term follow-up of pharmacological interventions.

## 6 Conclusion

In summary, this study delineates the clinical profile of AChR-Ab^+^/Titin-Ab^+^ MG patients in Southwest China. Our findings particularly the observed association between dual AChR/Titin positivity, greater disease severity, and higher rates of thyroid dysfunction, primarily hypothyroidism. These results suggest that MG patients presenting with severe symptoms or rapid disease progression at admission necessitate comprehensive Titin-Ab testing, enhanced screening for thyroid dysfunction, and close monitoring for respiratory muscle involvement.

## Data Availability

The original contributions presented in the study are included in the article/supplementary material, further inquiries can be directed to the corresponding authors.
